# Academic motivation in adolescents: the role of parental autonomy support, psychological needs satisfaction and self-control

**DOI:** 10.3389/fpsyg.2024.1384695

**Published:** 2024-05-10

**Authors:** Oğuzhan Çelik

**Affiliations:** ^1^Erzincan Binali Yildirim University, Erzincan, Türkiye; ^2^Erzincan University, Erzincan, Türkiye

**Keywords:** academic motivation, parental autonomy support, psychological needs, self-control, adolescent

## Abstract

**Background:**

Various perspectives on the existence and degree of motivation, which is a crucial factor influencing human behavior, have been studied for many years. Especially in adolescence, a phase marked by rapid change, motivation plays a crucial role in supporting young people to achieve their goals, fulfill their responsibilities, and experience healthy development.

**Aims:**

The present study aims to investigate the structural relationships among perceived parental autonomy support, satisfaction of psychological needs, self-control, and academic motivation.

**Sample:**

The study was conducted with a total of 427 high school students, including 230 females and 197 males, aged between 14 and 19 (M = 15.82; SD = 1.02).

**Method:**

Participants completed self-report measures of academic motivation, parental autonomy support, psychological needs satisfaction, and self-control. Structural equation modeling was performed to explore complex relationships between variables of interest.

**Results:**

The study revealed that psychological needs satisfaction increased as parental autonomy support increased, and self-control also increased with the satisfaction of psychological needs. It was also found that satisfaction of psychological needs was positively related to both intrinsic and extrinsic motivation. Additionally, self-control was associated with increased intrinsic motivation and decreased amotivation. In addition, it was found that parental autonomy support has an indirect relationship with academic motivation through the satisfaction of psychological needs and self-control.

**Conclusion:**

It is believed that these connections will lead to a deeper understanding of the significant processes in adolescence and serve as a foundation for developing and implementing psycho-educational interventions related to these variables.

## Introduction

From one standpoint, there exists an assertion that motivation is an individualistic trait found in some individuals but not in others. Conversely, an alternate viewpoint posits that while motivation is universally present in every individual, encompassing living entities, the degrees of motivation differ among them. In certain organisms, motivation can be notably low to the extent that they are labeled as “amotivation.” According to Tolman’s (1951) definition, motivation is delineated as a purposeful, predetermined, and goal-oriented behavior, involving specific forces that influence the individual in initiating, sustaining, and directing behavior. In this context, motivation can be succinctly characterized as the inclination to engage in an activity. It is anticipated that the intensity of this inclination correlates with the fulfillment of the individual’s needs through the execution of the action.

### Self-determination theory and motivation during adolescent

Self-determination theory (SDT) centers on human motivation, development, and well-being, with an emphasis on motivation types rather than quantity (Deci and Ryan, 1985). SDT posits three distinct motivation types based on the interplay between needs and the environment: intrinsic motivation, extrinsic motivation, and amotivation. Intrinsic motivation entails participating in activities for pleasure, personal benefit, or alignment with personal values, in contrast to being motivated by rewards, social pressure, or guilt avoidance (Ryan and Deci, 2017). Extrinsic motivation, on the other hand, comprises factors such as the desire for approval, avoidance of shame, and conditional self-esteem, arising from both the prospect of receiving rewards or escaping punishment (Deci and Ryan, 2008). When individuals perceive external control, they experience pressure to conform in thought, emotion, or action, labeled as extrinsic motivation. This motivation surfaces when the completion of an activity is deemed necessary for a desired outcome. Both intrinsic and extrinsic motivation fuel an individual’s energy to initiate action, contrasting with amotivation, which denotes a lack of motivation. Amotivation arises when there is no intention or impulse to sustain an activity due to a perceived lack of value, feelings of inadequacy, or the belief that the desired outcome is unattainable (Ryan and Deci, 2000). It is posited that these motivation types demonstrate a progression from amotivation to extrinsic motivation and later to intrinsic motivation (Deci and Ryan, 2000). According to this notion, individuals are initially amotivated, then learn to be externally motivated, and subsequently discover intrinsic motivation, thereby exhibiting a consistent will aligned with pleasure, personal benefit, or personal values.

SDT suggests that individuals possess an inherent drive for arousal and learning from birth (Deci and Ryan, 1985). This inherent motivation undergoes changes throughout life, especially during adolescence, where the natural motivations for learning become a notable strength for teenagers. According to Yeager et al. (2017), a teenager naturally motivated to learn initially directs their attention in the learning environment towards identifying what is essential for gaining status (extrinsic motivation). Subsequently, these behaviors may become routine, transforming into intrinsic motivation, characterized by a consistent will aligned with pleasure, personal benefit, or personal values.

Considering the remarkable changes in adolescence and the increased quest for independence motivation plays a crucial role during this period, supporting teenagers in setting their own goals, taking on responsibilities, and making independent decisions (Pickhardt, 2013). It also propels adolescents to refine social skills, establish friendships, and foster positive relationships with their families (Medeiros et al., 2021). Furthermore, motivation positively influences the emotional and mental well-being of adolescents, empowering them to enhance coping skills when facing challenges (De France and Hollenstein, 2021). Motivation also aids teenagers in understanding their values, exploring their passions, and forming their personal identities (Branje et al., 2021). Additionally, adolescence entails the determination of educational and career goals. Motivation empowers individuals to exert effort towards these goals, with academic motivation playing a pivotal role in activities such as studying, focusing on learning and investing effort in future careers (Hirschi and Vondracek, 2009). A meta-analysis conducted by Owen et al. (2014), determined a weak to moderate positive relationship between the motivation levels of adolescents and their overall performance. Additionally, it was found that intrinsic motivation was moderately positively related, extrinsic motivation was weakly positively related, and amotivation was weakly negatively related to overall performance in adolescents. Furthermore, motivation is linked not only to academic performance but also to the mental well-being of adolescents. For instance, research indicates that adolescents exhibiting elevated levels of motivation tend to experience greater life satisfaction (Luhmann and Hennecke, 2017) and reduced incidence of mental health problems (Strobel et al., 2021; Vella et al., 2021).

Considering the importance of motivation on adolescents’ well-being, revealing the individual and environmental factors having a role in the development of motivation. Therefore, exploring how familial traits influence the development of motivation in adolescents may empower them to surmount obstacles to motivation development. Consequently, this may reduce the probability of adolescents experiencing low motivation and mental health challenges (Strobel et al., 2021). Considering the connection between motivation and resilience (Trigueros et al., 2019), it can be posited that adolescents with high levels of motivation are better prepared to confront difficulties and maintain motivation in adverse situations. In conclusion, the recognition of personal and familial elements that impact motivation is essential for enhancing motivation in adolescents, nurturing their overall growth, and alleviating potential risk factors.

Deci and Ryan (1985) propose that the manifestation of learning motivation within the natural drive is contingent upon satisfying an individual’s psychological needs. In simpler terms, motivation is contingent upon meeting these needs.

### The role of parental autonomy support, basic psychological needs and self-control

According to SDT, there are three interrelated psychological needs: autonomy, representing engaging in behavior willingly and independently; competence, signifying effective action experience; and relatedness, denoting a sense of connection with significant others (Deci and Ryan, 2000). Competence needs entail the desire to feel capable of achieving goals, autonomy needs express the necessity for self-driven behaviors, and relatedness needs involve the need for acceptance by significant others such as parents, siblings, peers, and teachers (Deci and Ryan, 1985). Individuals strive to fulfill basic psychological needs, and the extent to which these needs are met may influence both the type and intensity of motivation in a specific activity (Deci and Ryan, 2008). In line with the SDT, Kalajas-Tilga et al. (2020) also underscore the impact of psychological needs on motivation by revealing a positive correlation between psychological need satisfaction and intrinsic motivation in adolescents.

As per Deci and Ryan (2000), intrinsic motivation hinges on how well an individual’s perceived competence, autonomy, and relatedness needs are satisfied in their social environment. This perspective aligns with SDT’s core principle, emphasizing the impact of social factors, particularly attitudes and behaviors of significant others like parents and teachers, on motivation (Ryan and Deci, 2017). Supporting this notion, Buzzai et al. (2020) demonstrated the relationship between need-supportive interpersonal behaviors and well-being, dispositional optimism, positive affectivity, and adaptive explanatory style. Recent studies also showed a positive correlation between social support from significant others, resilience, academic motivation, and academic achievement (Li et al., 2020; Lin et al., 2024). Considering the developmental trajectory of motivation from amotivation to extrinsic motivation and eventually to intrinsic motivation (Deci and Ryan, 2000), it can be anticipated that individuals commence tasks with a lack of motivation and gradually learn extrinsic motivation, influenced by their immediate environment, notably their parents. Ultimately, they are expected to develop intrinsic motivation.

The significance of fulfilling individuals’ psychological needs through close relationships and the gradual development of motivation within a process emphasize the role of parental attitudes and behaviors. This notion aligns with the research findings of Haerens et al. (2015), where adolescents receiving autonomy support from significant others demonstrated the ability to choose their path to achieve goals, held more positive beliefs in skill development, and felt valued by important individuals. Similarly, a meta-analysis by Hagger and Chatzisarantis (2016) supported these findings, showing that parents’ autonomy support behaviors positively influenced adolescents’ autonomy, competence, and relatedness needs, subsequently enhancing their performance. Several studies found that perceived autonomy support from teachers, parents, and peers contributes to adolescents’ psychological need satisfaction, correlating with increased intrinsic motivation (e.g., Vierling et al., 2007; Standage et al., 2012; Wang, 2017).

Parental autonomy support attitudes encompass skills such as understanding, communication, inclusion, challenge, encouragement, modeling, development, coaching, feedback reception, and providing fair rewards (Cook, 1991). A parenting attitude that includes these skills is expected to positively influence the motivation processes of adolescents. This notion is supported by Wang (2017), emphasizing that autonomy support from parents enhances intrinsic motivation through the satisfaction of fundamental psychological needs. Du et al. (2023) also highlighted that parental autonomy support increases grit by enhancing fundamental psychological needs, subsequently leading to increased motivation. Similarly, autonomy support from both mother and father has been found to support the satisfaction of fundamental psychological needs, reduce the thinness drive, and increase motivation (Barbierik et al., 2022). These findings may demonstrate that autonomy support from parents supports psychological need satisfaction and enhances motivation by fostering various skills.

Recent research indicates that a parent’s behaviors supporting autonomy in adolescents might also contribute to the development of self-control (Li et al., 2019; Wang Q. et al., 2022). These studies also show that negative parenting styles, such as excessive punishment and helicopter parenting, adversely affect self-control. Bernier et al. (2010) found that autonomy support in the 1–3-year period, much like adolescence, is the most robust predictor of developing self-control skills in newly walking children. Considering that parents who support adolescent autonomy guide them to solve their problems, set clear behavioral standards, gradually internalize social norms, and develop an understanding of appropriate behavior (Özdemir et al., 2013), this attitude is expected to fulfill both the fundamental psychological needs of adolescents and support the development of self-control skills.

When considering the behaviors of experiencing behavior willingly and independently, performing actions effectively, and feeling connected to significant others at the core of fundamental psychological needs (Deci and Ryan, 2000), the relationship with self-control becomes better understood. Considering that self-control involves the ability to regulate one’s thoughts, feelings, and behaviors to adhere to social values, moral norms, and pursue long-term goals (Baumeister et al., 2007), it can be suggested that self-control may be nourished by autonomy, competence, and relatedness traits. Additionally, adolescents with high autonomy and competence may tend to have a strong sense of responsibility and place greater importance on achieving long-term goals, indicating stronger self-control abilities (Dreves and Blackhart, 2019). Furthermore, adolescents with high self-control may exhibit better adaptation to situations and environments and have more advanced interpersonal skills (Tangney et al., 2004), serving as an indicator that they satisfy their fundamental psychological needs.

Vohs and Heatherton (2000) defined self-control as the ability to override, inhibit, or alter impulses using cognitive and attentional resources to achieve personal goals, drawing attention to the need for power or energy in this process. In parallel with this idea, the Self-Control Model (Baumeister and Heatherton, 1996) states that individuals’ self-control capacity is a limited resource that increases, decreases, or becomes depleted over time. It is believed that this is one reason why people’s energy depletes more rapidly on days when they engage in activities that require self-control, such as learning. This is because learning requires possessing self-control skills, such as completing tasks on time, preventing leisure activities from hindering learning, effectively managing study time, selecting appropriate courses, and preventing distractions from harming learning (Tangney et al., 2004). Thus, it may be argued that perceiving support from others and meeting psychological needs help to increase self-control, which in turn motivates the individual to act. This idea is supported by the notion that adolescents with high self-control tend to achieve better grades (Tangney et al., 2004). Essentially, the exercise of self-control can amplify academic motivation by enhancing an adolescent’s academic performance. Similarly, Duckworth et al. (2019) demonstrated that self-control enhances school performance. Moreover, Bargh and Chartrand (1999) suggested that an individual lacking self-control would give up the struggle and not adhere to strict rules when experiencing disappointment in any situation. Instead, they would exhibit automatic behaviors to bypass the process. Particularly, there could be a connection between giving up the struggle in a situation and motivation, as the source of energy needed for the struggle is motivation (Ryan and Deci, 2017).

Based on all these findings, it can be said that perceived autonomy support from parents is related to academic motivation. In addition, research shows that some variables affect the relationship between perceived parental autonomy support and academic motivation. In the literature, it is emphasized that autonomy support perceived from parents supports adolescents’ basic psychological needs such as making their own decisions and taking responsibility by strengthening their sense of independence and self-confidence. Considering that such skills are an important tool for achieving goals, the relationship between basic psychological needs and motivation is better understood. In addition, it can be thought that autonomy and belief in one’s own abilities will also support the level of self-control. It is expected that self-controlled individuals will control the situations that will affect themselves negatively, participate more in the learning process, and show more interest and effort in learning. It can be said that this situation will support academic motivation. Therefore, it is thought that an autonomy-supportive environment provided by parents to their children can increase academic motivation by supporting self-control skills, as well as meeting the psychological needs of adolescents. This situation can contribute to adolescents to grow up more successful, happy and healthy.

### The present study

**Figure 1:**
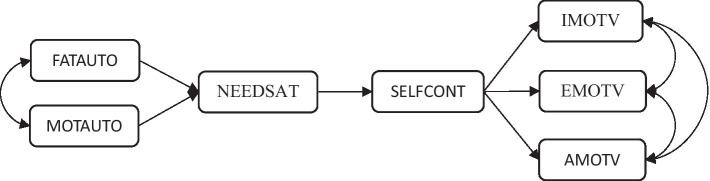
Hypothesized structural model. MOTAUTO, Mother Autonomy Granting; FATAUTO, Father Autonomy Granting; NEEDSAT, Need Satisfaction; SELFCONT, Self-Control; IMOTV, Intrinsic Motivation; EMOTV, Extrinsic Motivation; AMOTV, Amotivation.

Considering the literature summarized above, it can be argued that there is an interaction between parental behaviors that support autonomy, satisfaction of psychological needs, self-control, and academic motivation. Existing literature includes studies investigating the relationship between adolescents’ academic motivation levels and various variables such as physical performance (Knittle et al., 2018), school commitment, perceptions of the school environment, gender, and ethnicity (Wang and Eccles, 2013), and parental and family structure (Lau et al., 2007; Wang H. et al., 2022). Conversely, there might be a requirement for research endeavors aimed at elucidating the psychological processes that underlie how parental traits and personal factors impact the formation of motivation in adolescents. Therefore, this study seeks to contribute to comprehending the intricate interaction between familial and individual factors associated with motivation, shedding light on the underlying mechanisms of motivation development in adolescents. Aligned with this overarching goal, the study aims to scrutinize the structural relationships among perceived autonomy support from parents, satisfaction of psychological needs, self-control, and academic motivation, as delineated in the hypothetical model presented in Figure 1. According to this model, parental autonomy support is posited to enhance adolescents’ self-control by satisfying their psychological needs. Concurrently, an augmentation in self-control is theorized to elevate both internal and external motivation while diminishing amotivation.

## Method

### Participants

A total of 548 Turkish adolescents were recruited through online convenience sampling for the study. In the measurement battery, a “checking question” with the statement “If you are reading this question, please tick “3″“was used, resulting in the exclusion of 121 participants who did not provide the correct answer. Consequently, the final sample comprised 427 participants, with ages ranging from 14 to 19 (M = 15.82; SD = 1.02). The gender distribution of the sample was 53.9% female (*n* = 230) and 46.1% male (*n* = 197). The distribution across school grades was as follows: 9th grade (21.5%), 10th grade (30.4%), 11th grade (33.3%), and 12th grade (14.8%). All participants were briefed on the study’s objectives and procedures and provided written informed consent. The study’s purpose and procedures received approval from the local university’s ethical committee.

### Instruments

#### Academic motivation scale (AMS)

The Academic Motivation Scale (AMS), developed by Vallerand et al. (1992), comprises 28 items, with students responding to the prompt “What are your reasons for pursuing higher education?” The AMS encompasses seven subscales: amotivation (e.g., “I once had good reasons for going to college; however, now I wonder whether I should.”), external regulation (e.g., “because with only a high-school degree I would not find a high paying job later on.”), introjected regulation (e.g., “to show myself that I am an intelligent person.”), identified regulation (e.g., “because this will help me make a better choice regarding my career orientation.”), intrinsic motivation to know (e.g., “because I experience pleasure and satisfaction while learning new things.”), intrinsic motivation to experience stimulation (e.g., “for the pleasure that I experience when I read interesting authors.”), and intrinsic motivation to accomplish (e.g., “for the pleasure that I experience while surpassing myself in my studies.”). Responses to the items are rated on a scale from one (indicating no correspondence) to seven (indicating complete correspondence). Each subscale includes four items, resulting in subscale scores that can range from 4 to 28. A high score on a subscale signifies a strong endorsement of the corresponding academic motivation. The original study of the BSCS initially identified it as a scale with seven factors. However, subsequent research has indicated that models with one, three, five, and seven factors all demonstrated adequate model fit (Barkoukis et al., 2008; Stover et al., 2012). The internal consistency of the AMS’s seven sub-scales was deemed satisfactory, with Cronbach’s alphas ranging from 0.62 to 0.86. Karagüven-Ünal (2012) adapted the AMS for use in Turkish and found that Cronbach’s alphas ranged from 0.67 to 0.87. In the current study, the Cronbach’s Alphas ranged from 0.53 to 0.86 (Table 1).

**Table 1:** Means, standard deviations, internal reliability, and pearson product–moment correlation coefficients.

	1	2	3	4	5	6	7	8	9	10	11	12	13	14	15	16
1. MOTAUTO	–															
2. FATAUTO	0.470**	–														
3. NEEDSAT	0.355**	0.339**	–													
4. AUTS	0.372**	0.357**	0.836**	–												
5. RELS	0.246**	0.182**	0.695**	0.378**	–											
6. COMPS	0.223**	0.258**	0.827**	0.586**	0.320**	–										
7. SELFCONT	0.237**	0.229**	0.459**	0.402**	0.233**	0.436**	–									
8. SDISCP	0.211**	0.201**	0.487**	0.424**	0.240**	0.470**	0.893**	–								
9. SIMPLS	0.212**	0.207**	0.332**	0.293**	0.176**	0.307**	0.892**	0.592**	–							
10. IMKNWL	0.240**	0.145**	0.321**	0.318**	0.187**	0.251**	0.304**	0.266**	0.276**	–						
11. IMACCMP	0.248**	0.152**	0.347**	0.352**	0.155**	0.304**	0.281**	0.290**	0.211**	0.682**	–					
12. IMSTIM	0.234**	0.146**	0.262**	0.283**	0.103*	0.226**	0.258**	0.247**	0.213**	0.772**	0.651**	–				
13. IDTREG	0.096*	0.119*	0.226**	0.191**	0.158**	0.185**	0.225**	0.205**	0.197**	0.449**	0.555**	0.333**	–			
14. INTRREG	0.148**	0.085	0.226**	0.233**	0.073	0.217**	0.076	0.093	0.043	0.559**	0.736**	0.548**	0.380**	–		
15. EXTREG	0.056	0.092	0.161**	0.149**	0.082	0.146**	0.008	0.044	−0.029	0.391**	0.494**	0.283**	0.582**	0.531**	–	
16. AMOTV	−0.162**	−0.167**	−0.273**	−0.230**	−0.210**	−0.208**	−0.364**	−0.297**	−0.353**	−0.374**	−0.345**	−0.241**	−0.419**	−0.146**	−0.093	–
Mean	18.59	14.05	43.00	13.95	15.11	13.93	40.98	23.38	17.59	17.23	17.71	14.78	21.85	16.29	19.49	14.05
Sd	4.55	3.62	8.09	3.41	3.15	3.68	8.91	5.00	4.98	6.61	6.33	6.32	6.06	6.35	5.22	7.14
Skewness	−0.895	−0.635	−0.240	−0.215	−0.394	−0.493	−0.094	−0.156	−0.004	−0.159	−0.257	0.167	−0.948	−0.044	−0.392	0.314
Kurtosis	0.674	0.252	−0.253	−0.377	−0.436	−0.137	−0.079	0.026	−0.392	−0.775	−0.667	−0.621	0.243	−0.744	−0.123	−0.910
α	0.81	0.71	0.82	0.71	0.65	0.77	0.81	0.69	0.72	0.83	0.78	0.78	0.86	0.74	0.53	0.82

* *p* < 0.05, ** *p* < 0.01; MOTAUTO, Mother Autonomy Granting; FATAUTO, Father Autonomy Granting; NEEDSAT, Need Satisfaction; AUTS, Autonomy Satisfaction; RELS, Relatedness Satisfaction; COMPS, Competence Satisfaction; SELFCONT, Self-Control; SDISCP, Self-Discipline; SIMPLS, Impulse Control; IMKNWL, Intrinsic Motivation to Know; IMACCMP, Intrinsic Motivation toward Accomplishment; IMSTIM, Intrinsic Motivation to Experience Stimulation; IDTREG, Identified Regulation; INTRREG, Introjected Regulation; EXTREG, Extrinsic Regulation; AMOTV, Amotivation.

#### Brief self-control scale (BSCS)

The BSCS was created to assess the level of self-control and comprises 13 items rated on a 5-point Likert scale, ranging from 1 (not at all true of me) to 5 (totally true of me) (Tangney et al., 2004). While the initial development study of the BSCS identified it as a single-factor scale, subsequent research has suggested that it actually encompasses two factors (Fulford et al., 2008; Maloney et al., 2012). The total score on the BSCS is calculated by summing the responses to all 13 items, with higher scores indicating greater levels of self-control. The BSCS has demonstrated strong internal consistency, as evidenced by a Cronbach’s alpha of 0.85 (Tangney et al., 2004). Nebioglu et al. (2012) adapted the BSCS to Turkish and suggested that the BSCS had two factors: self-discipline (e.g., “I am good at resisting temptation”) and impulsivity (e.g., “I often act without thinking through all the alternatives”). The Turkish version of the BSCS yielded acceptable internal consistency, with Cronbach’s alphas of α = 0.81 for self-discipline, α = 0.87 for impulsivity, and α = 0.83 for total score. As seen in Table 1, Cronbach’s Alphas ranged between 0.69 to 0.81 in the present study.

#### Leuven adolescent perceived parenting scale (LAPPS)

LAPPS was created by Soenens et al. (2004) to evaluate adolescents’ perceptions of their parents’ attitudes and behaviors. LAPPS comprises 28 self-report items, rated on a 5-point scale from 1 (never true) to 5 (always true), and encompasses four subscales: responsiveness, behavioral control, psychological control, and autonomy support. In this study, only the autonomy granting subscale was utilized to gauge adolescents’ perceptions of their parents’ autonomy granting. Participants completed LAPPS for both their mothers and fathers. The internal consistency coefficients of LAPPS’s subscales ranged from 0.76 to 0.90 for the mother version and 0.71 to 0.91 for the father version. The Turkish version of LAPPS, adapted by Sevim (2014), includes 21 items for the mother version and 19 items for the father version. The LAPPS for both mother and father encompasses four subscales: sensitivity (e.g., “She/He often smiles at me.”), behavioral control (e.g., “She/He gives me as much freedom as I want.”), psychological control (e.g., “If I do something she/he does not like, she/he gets cold and snippy with me.”), autonomy granting (e.g., “She/he allows me to make my own decisions in matters that concern me.”). The autonomy support subscale comprises four items for the father version and five items for the mother version. The Turkish version of LAPPS confirmed the original four-factor structure and demonstrated acceptable internal consistency, with Cronbach’s alphas ranging from 0.58 to 0.88 for the mother version and 0.67 to 0.91 for the father version. As indicated in Table 1, the Cronbach’s alpha coefficient for the autonomy granting subscale was 0.81 for the mother form and 0.71 for the father form in the present dataset.

#### Basic psychological need satisfaction and frustration scale (BSNSFS)

Chen et al. (2015) developed the BSNSFS to evaluate individuals’ satisfaction and frustration regarding their basic psychological needs. The scale comprises 24 self-report items, with participants responding on a five-point Likert scale ranging from 1 (Not true at all) to 5 (Completely true). The scale produces scores on six sub-scales, three of which assess satisfaction of the basic psychological needs (autonomy [e.g., “I feel my choices express who I really am”], relatedness [e.g., “I feel that the people I care about also care about me”], and competence [e.g., “I feel capable at what I do”]), while the remaining three measure frustration of these needs (autonomy [e.g. “Most of the things I do feel like “I have to.”], relatedness [e.g., “I feel the relationships I have are just superficial”], and competence [e.g., “I feel insecure about my abilities”]). In this particular study, three sub-scales focusing on need satisfaction were utilized. The internal consistency of the BSNSFS’s six sub-scales was found to be satisfactory, with Cronbach’s alphas ranging from 0.64 to 0.89. Additionally, composite scores for need satisfaction and frustration may have been computed (Kindt et al., 2016). The Turkish adaptation of the BSNSFS, conducted by Selvi and Bozo (2020), demonstrated high internal consistency and maintained the original factor structure, with Cronbach’s alphas ranging from 0.74 to 0.88. The Turkish version of the BSNSFS exhibited favorable psychometric properties in the present study, with Cronbach’s alphas of α = 0.82 for autonomy satisfaction, *α* = 0.82 for relatedness satisfaction, and *α* = 0.83 for competence satisfaction (Table 1).

### Data analysis

The data analysis was conducted in a two-step process. Firstly, descriptive statistics were computed, followed by testing a specified structural model to investigate the relationships among the variables of interest. The statistical analyses were performed using MPlus version 8.4 (Muthén and Muthén, 1998–2017) and JASP version 0.16.1 (JASP Team, 2024). Normality was assessed using skewness and kurtosis, and descriptive statistics and correlation coefficients were computed for the sample. A structural equation model (SEM) was then constructed to explore the relationships between the variables of interest. In the SEM, observed indicators of latent variables included the items of the “father autonomy support,” “mother autonomy support,” and “amotivation” subscales, as well as the subscales of the BSCS. Additionally, observed variables comprised the subscales of the BSNSFS focusing on need satisfaction, the subscales of the AMS evaluating intrinsic motivation, and extrinsic motivation. Following Anderson and Gerbing (1988) two-step SEM procedure, the measurement model was initially tested using a confirmatory factor analysis, and subsequently, the final structural model was examined. Model fit was assessed using the CFI (≥ 0.90), TLI (≥ 0.90), SRMR (≤ 0.08), and RMSEA (≤ 0.08) with a 90% CI, as recommended by Brown (2015), Kline (2011), and Wen et al. (2004). Furthermore, indirect effects were examined using the bootstrapping method with 2000 bootstrap samples and a 95% bias-corrected confidence interval.

## Findings

### Descriptive statistics

The statistical measures, including means, standard deviations, and Cronbach’s alphas for the psychometric instruments, as well as the correlations between scale scores, are detailed in Table 1.

### Measurement model

The measurement model did not demonstrate an acceptable fit to the data (χ^2^ (231) = 775.531, *p* < 0.001; RMSEA [90% confidence interval] = 0.074 [0.069–0.080], *p* < 0.05; CFI = 0.87; TLI = 0.85, and SRMR = 0.056). However, modification indices suggested a possible covariance between the error variances of the indicator variable pairs of IMSTIM-IMKNWL, EXTREG-IDTREG, MA18-MA12, FA16-FA7, and MOTV19-MOTV26. Since these items in each pair are semantically close and located in the same sub-dimension, a covariance between the error variance of two items in each pair was added to the model, and the CFA was performed again. The last CFA results demonstrated that the model fit the data well with the goodness-of-fit-indices as follows; *χ*^2^ (226) = 559.524, *p* < 0.001; RMSEA [90% confidence interval] = 0.059 [0.053–0.065], *p* < 0.05; CFI = 0.92; TLI = 0.91, and SRMR = 0.052. Each observed indicator exhibited significant loadings on its corresponding latent factor, with standardized factor loadings ranging from 0.455 to 0.925. Additionally, all latent variables displayed significant correlations with each other (*p* < 0.05), and the standardized correlational coefficients varied between 0.139 to 0.959.

### Structural model

We conducted a structural equation modeling analysis to investigate the theoretical relationships between parental autonomy support, psychological need satisfaction, self-control, and academic motivation. The model fit indices indicated that the proposed structural model was a good fit for the data, as evidenced by the following statistics: *χ*^2^ (234) = 570.388; *p* < 0.05; RMSEA [90% confidence interval] = 0.058 [0.052–0.064] *p* < 0.05; CFI = 0.921, TLI = 0.907, and SRMR = 0.054. The standardized regression coefficients indicated that the associations between self-control and extrinsic motivation, as well as the relationship between psychological need satisfaction and amotivation, were not statistically significant. Consequently, we removed direct paths between these variables from the model and subsequently re-analyzed the final model. The final structural model demonstrated a good fit with the data; *χ*^2^ (236) = 573.786; *p* < 0.05; RMSEA [90% confidence interval] = 0.058 [0.052–0.064] *p* < 0.05; CFI = 0.921, TLI = 0.908 and SRMR = 0.055.

### Direct relationships

**Figure 2:**
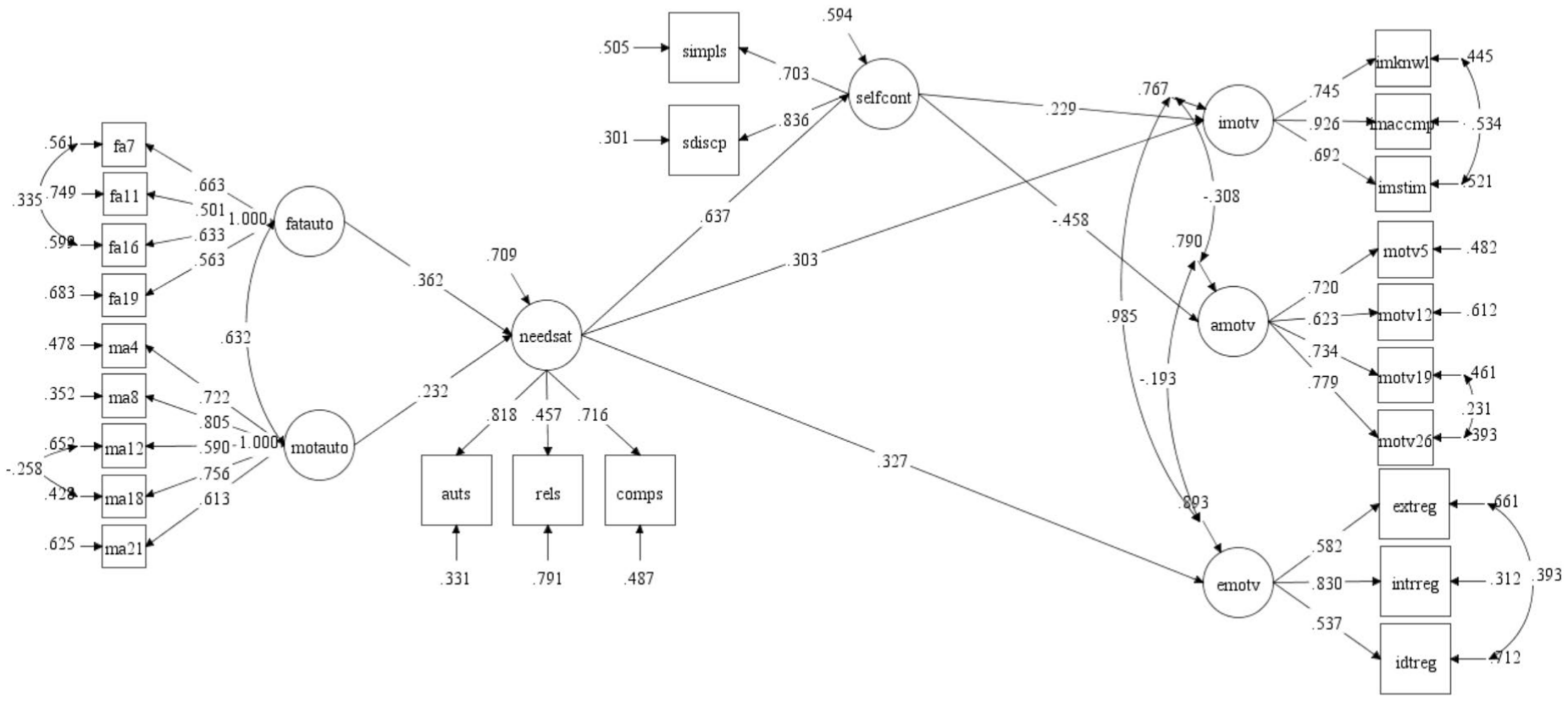
Standardized maximum likelihood estimates of the structural model. MOTAUTO, Mother Autonomy Granting, FATAUTO, Father Autonomy Granting; NEEDSAT, Need Satisfaction; AUTS, Autonomy Satisfaction; RELS, Relatedness Satisfaction; COMPS, Competence Satisfaction; SELFCONT, Self-Control; SDISCP, Self-Discipline; SIMPLS, Impulse Control; IMOTV, Intrinsic Motivation; IMKNWL, Intrinsic Motivation to Know; IMACCMP, Intrinsic Motivation toward Accomplishment; IMSTIM, Intrinsic Motivation to Experience Stimulation; EMOTV, Extrinsic Motivation; IDTREF, Identified Regulation; INTRREG, Introjected Regulation; EXTREG, Extrinsic Regulation; AMOTV, Amotivation. The diagram shows only significant estimates. All coefficients greater than 0.229 are significant at *p* ≤ 0.001. All coefficients greater than 0.160 are significant at *p* ≤ 0.05.

Following an evaluation of the fit indices of the structural model, an analysis was conducted on the direct relationships present within the structural model. Standardized regression coefficients showed that father autonomy granting (*β* = 0.362, SE = 0.099, *p* < 0.001) and mother autonomy granting (*β* = 0.232, SE = 0.095, *p* < 0.05) significantly contributed to psychological need satisfaction (Figure 2). Psychological need satisfaction positively correlated with self-control (*β* = 0.637, SE = 0.057, *p* < 0.001), intrinsic motivation (*β* = 0.303, SE = 0.068, *p* < 0.001) and extrinsic motivation (*β* = 0.327, SE = 0.063, p < 0.001). Additionally, there was a significant association between self-control and intrinsic motivation (*β* = 0.229, SE = 0.055, *p* < 0.001), as well as amotivation (*β* = −0.458, SE = 0.065, *p* < 0.001).

### Indirect relationships

The structural model demonstrated that all potential indirect relationships among the variables under investigation were statistically significant, as shown in Table 2. The total indirect effect of father autonomy granting intrinsic motivation was found to be statistically significant (*β* = 0.162, SE = 0.048, CI = 0.068–0.257, *p* < 0.001). Specifically, father autonomy granting had an indirect impact on intrinsic motivation through the psychological need satisfaction (*β* = 0.109, SE = 0.039, CI = 0.034–0.185, *p* < 0.05), and psychological need satisfaction and self-control in a sequential manner (*β* = 0.053, SE = 0.048, CI = 0.013–0.093, *p* < 0.05).

**Table 2:** Indirect relationships in the structural model.

Indirect relationships	Indirect *β (SE)*	95% BC-CI [LL, UL]	*t*	*p*-value
Total Indirect Effect from FATAUTO to IMOTV	0.162 (0.048)	[0.068, 0.257]	3.367	0.001
FATAUTO➔**NEEDSAT** ➔IMOTV	0.109 (0.039)	[0.034, 0.185]	2.837	0.005
FATAUTO➔**NEEDSAT**➔**SELFCONT**➔IMOTV	0.053 (0.053)	[0.013, 0.093]	2.581	0.010
Total Indirect Effect from MOTAUTO to IMOTV	0.104 (0.046)	[0.014, 0.194]	2.258	0.024
MOTAUTO➔**NEEDSAT** ➔IMOTV	0.070 (0.035)	[0.001, 0.139]	2.001	0.045
MOTAUTO➔**NEEDSAT**➔**SELFCONT**➔IMOTV	0.034 (0.017)	[0.001, 0.067]	2.005	0.045
FATAUTO➔**NEEDSAT**➔EMOTV	0.118 (0.041)	[0.039, 0.198]	2.911	0.004
MOTAUTO➔**NEEDSAT**➔EMOTV	0.076 (0.034)	[0.009, 0.143]	2.222	0.026
FATAUTO➔**NEEDSAT**➔**SELFCONT**➔AMOTV	−0.106 (0.036)	[−0.177, −0.034]	−2.910	0.004
MOTAUTO➔**NEEDSAT**➔**SELFCONT**➔AMOTV	−0.068 (0.030)	[−0.127, −0.009]	−2.247	0.025

The mediator variables are in bold. MOTAUTO, Mother Autonomy Granting; FATAUTO, Father Autonomy Granting; NEEDSAT, Need Satisfaction; SELFCONT, Self-Control; IMOTV, Intrinsic Motivation; EMOTV, Extrinsic Motivation; AMOTV, Amotivation. *Β*, Standardized regression coefficient; SE, Standard error; BC-CI, Bias-corrected confidence intervals. LL and UL indicate the lower and upper limit of a confidence interval, respectively.

The total indirect effect of maternal autonomy granting on intrinsic motivation was determined to be statistically significant (*β* = 0.104, SE = 0.046, CI = 0.014–0.194, *p* < 0.05). It was found that psychological need satisfaction acts as a mediator in the relationship between maternal autonomy granting and intrinsic motivation (*β* = 0.070, SE = 0.035, CI = 0.001–0.139, *p* < 0.05). Maternal autonomy granting was observed to have an indirect impact on intrinsic motivation through psychological need satisfaction and self-control (*β* = 0.034, SE = 0.017, CI = 0.001–0.067, *p* < 0.05).

Father autonomy granting (*β* = 0.118, SE = 0.041, CI = 0.039–0.198, *p* < 0.05) and mother autonomy granting (*β* = 0.076, SE = 0.034, CI = 0.009–0.143, *p*< 0.05) related to extrinsic motivation through psychological need satisfaction. Additionally, the relationship between amotivation and both father autonomy granting (*β* = −0.106, SE = 0.036, CI = −0.177–0.034, p < 0.05) and mother autonomy granting (*β* = −0.068, SE = 0.030, CI = −0.127- -0.009, *p* < 0.05) is serially mediated by psychological need satisfaction and self-control.

## Discussion

This study demonstrates a positive association between increased parental autonomy support and the satisfaction of psychological needs, leading to enhanced self-control. This study demonstrates a positive relationship between increased parental autonomy support and the satisfaction of psychological needs, which is also associated with enhanced self-control. Additionally, it reveals a positive correlation between the satisfaction of psychological needs and both intrinsic and extrinsic motivation. Moreover, increased self-control is found to be associated with heightened intrinsic motivation and reduced amotivation. Beyond these findings, the study unveils an indirect relationship between parental autonomy support and academic motivation through the mediation of psychological need satisfaction and self-control. As per this research, adolescents perceiving autonomy support from their parents exhibit greater proficiency in meeting their psychological needs, a pattern consistent with findings from a study conducted by Kalajas-Tilga et al. (2020).

The rationale behind this phenomenon may lie in adolescents feeling more socially autonomous and competent due to parental support, enabling them to excel in establishing social connections with others (Chen and Hypnar, 2015). The logic underlying this phenomenon could be attributed to the perception among adolescents experiencing parental support that they possess greater social autonomy and competence, consequently enhancing their efficacy in establishing social affiliations with peers (Chen and Hypnar, 2015). Parental autonomy support shapes adolescents to be more independent, competent, and relational, thereby fulfilling their basic psychological needs (Wang, 2017). Parental autonomy support can support adolescents’ basic psychological needs by enabling them to become more independent, competent and relational (Wang, 2017).

Prior research conducted among Turkish families has corroborated the findings of the present study. For example, Yılmaz and Büyükcebeci (2019) found that families exhibiting a more protective parental approach tend to limit their children’s autonomy. This perspective is further supported by the research conducted by Gök (2019), which also observed a negative impact on children’s autonomy within families displaying a protective parenting style. Gök’s study highlighted the interplay between subjective well-being, fundamental psychological needs, and parental support for autonomy, suggesting that reduced parental autonomy support may hinder children’s psychological well-being. Moreover, Kocayörük (2012) noted a decline in Turkish adolescents’ perception of autonomous self-management as parental autonomy support decreased.

Another plausible explanation for the impact of parental autonomy support on psychological needs is the increased parental involvement in extracurricular activities outside school (McDavid et al., 2012). Another plausible explanation for the impact of parental autonomy support on psychological needs could be increased parental involvement in extracurricular activities outside school (McDavid et al., 2012). Parents displaying a more autonomous attitude in such activities provide their children with more opportunities for socialization and physical activity. Adolescents with higher physical experience and competence are more popular among their peers, potentially fulfilling their relational needs. This notion is supported by a study conducted by Hong et al. (2012), emphasizing that authoritarian or excessively controlling traditional parenting styles fail to fulfill the psychological needs of adolescents.

Another noteworthy discovery of the current research is that the satisfaction of psychological needs positively predicts self-control. In simpler terms, individuals who can satisfy their psychological needs demonstrate more effective control over their emotions, thoughts, and behaviors. In other words, individuals who can meet their psychological needs tend to have greater control over their emotions, thoughts, and behaviors. The positive relationship between self-control and adaptive, positive behaviors has been highlighted in numerous studies (Orkibi et al., 2014; Kerret et al., 2016; Orkibi and Ronen, 2017). Especially during the tumultuous years of adolescence, adolescents who can satisfy their psychological needs are expected to enhance their self-control skills. Orkibi and Ronen (2017) conducted a study involving 1,576 adolescents around the age of 16, examining SDT in the educational context, and indicated a positive relationship between self-control and perceived satisfaction of psychological needs. Adolescents exhibiting a high degree of self-control are expected to respond to life challenges more deliberately and constructively. This mindset is believed to contribute to adolescents’ perceptions of their psychological needs being met. This interpretation aligns with research conceptualizing self-control as a skill enabling individuals to act voluntarily in pursuit of their goals (autonomy), overcome challenges, and consequently feel more competent and capable (competence) (Rosenbaum, 1990; Rosenbaum and Ronen, 2013).

In a study by Gilbert et al. (2023) involving approximately 2000 university students, it was reported that students with higher psychological need satisfaction exhibited superior self-control performance. When self-control is considered the ability to regulate unwanted thoughts, emotions, and behaviors and initiate desired ones (Baumeister and Heatherton, 1996), individuals possessing these skills are expected to be more autonomous, more competent, and more relationally oriented. Having autonomy in determining one’s own goals and standards, coupled with competence in achieving these goals and standards, may support the development of adolescents’ self-control. This notion broadly aligns with the definition of self-control encompassing both conscious and unconscious processes underlying any behavior guided by goals or standards (Baumeister, 2002). In other words, behaviors that individuals construct with their own will and find more competent are easier to accept and control, contributing to both the satisfaction of psychological needs and the development of self-control skills.

Another finding of this research is the positive prediction of intrinsic motivation by self-control and the negative prediction of amotivation. In simpler terms, individuals with high self-control exhibit high levels of intrinsic motivation and low levels of amotivation. This finding aligns with the idea from SDT that motivational types in human life progress from amotivation to extrinsic motivation and eventually to intrinsic motivation (Deci and Ryan, 2000). In this context, the idea that an adolescent’s innate academic motivation (Deci and Ryan, 1985) gradually evolves towards intrinsic motivation could explain this finding. Especially in the academic domain, adolescents with high self-control, who can regulate their behaviors in response to external pressures or rewards, choose their behaviors with their own will, and perform them for their own enjoyment and preferences, experience an elevation in intrinsic motivation (Ryan and Deci, 2006). Conversely, such pressures may turn overwhelming and coercive, limiting an adolescent’s control over their own world (Baym, 2015).

This limitation is expected to diminish the adolescent’s internal motivation, the energy source, leading to demotivation over time. The consistency of this interpretation is evident in the negative impact of social pressure on autonomy and competence, reducing self-control (Halfmann and Rieger, 2019). The inability to act autonomously and achieve personal preferences may result in the adolescent feeling insufficient and being unable to assume control over their behavior, consequently leading to an energy deficit known as demotivation. Moreover, the concept of self-control, which can be articulated as an individual’s ability to manage their behaviors and preferences, appears to be closely associated with intrinsic motivation. This idea is consistent with the observation that individuals may experience a decrease in motivation levels when faced with cognitive overload, making it challenging to rectify or regulate their thoughts, as noted by Gilbert et al. (1993). Similarly, Wilson and Brekke (1994) asserted that individuals with high cognitive load, but strong cognitive adjustments or self-regulation exhibit elevated motivation levels.

Finally, in this study, it was determined that the relationship between parental autonomy support and motivation is mediated by the satisfaction of psychological needs and self-control. According to this finding, parental autonomy support plays an intermediary role in the satisfaction of psychological needs between parental autonomy support and both intrinsic and extrinsic motivations. This suggests that when adolescents perceive autonomy support from their parents, their intrinsic and extrinsic motivations increase when their psychological needs are satisfied. This finding is consistent with Wang (2017) study, which indicated that the satisfaction of psychological needs mediated the relationship between motivation and autonomy support from parents. One plausible interpretation of this situation involves considering the impact of the relationship between competence and relatedness on extrinsic motivation. For instance, the attainment of external motivation, such as peer acceptance, often relies on adolescent excelling in something highly esteemed by their peers (Evans and Roberts, 1987), particularly observed in boys. A male adolescent demonstrating competence in an area highly valued by peers receives increased external reinforcement, thereby experiencing higher external motivation (Standage et al., 2003). Autonomy-supportive behavior received from parents contributes to an enhancement in the adolescent’s competence, thereby positively influencing both intrinsic and extrinsic motivations. Positive feedback from others, in the form of rewards, is perceived as indicators of success, fostering psychological need satisfaction (Deci et al., 1999).

Another perspective suggests that adolescent demonstrating autonomy support behavior from their parents may bolster their sense of autonomy, resulting in an augmented social competence and increased interpersonal connections. Parental autonomy support behaviors, positively linked to psychological need satisfaction (Zhang et al., 2011), can encourage adolescents to establish relationships. Conversely, an increase in parental control is anticipated to diminish psychological need satisfaction. Especially due to the influence of traditional family culture, many parents in countries like Turkey excessively worrying about their children can hinder their autonomy. This situation may lead adolescents to exhibit behavior in the direction of impaired relatedness from their psychological needs, resulting in an inability to find the energy required for such behavior, leading to demotivation and dependency (Ngan-ling Chow and Zhao, 1996). This situation can lead adolescents to exhibit behaviors towards detachment stemming from their psychological needs, inability to find the required energy for such behaviors, affecting motivation and dependency (Ngan-ling Chow and Zhao, 1996). This perspective aligns with the concept in SDT that emphasizes autonomous behavior being conducted with a sense of choice (Deci and Ryan, 2008). A dependent adolescent cannot make free choices, and an adolescent unable to make free choices may fail to satisfy their need for relatedness, consequently lacking the necessary motivation for relational behaviors. Therefore, the autonomy-non-supportive attitudes of parents may potentially lead to low intrinsic and extrinsic motivation in adolescents as their psychological needs remain unmet. Therefore, the autonomy-non-supportive attitudes of parents may influence potentially low intrinsic and extrinsic motivation in adolescents whose psychological needs are not met.

According to the research findings, there is also a mediating role of the satisfaction of psychological needs and self-control in the relationship between parental autonomy support and intrinsic motivation and amotivation. This finding suggests that parental autonomy support not only fulfills psychological needs but also leads to an increase in self-control, subsequently bolstering intrinsic motivation and diminishing amotivation. The relationship between motivation and “effort expended to meet individual needs” (Greenberg, 1999) can explain the connection between motivation and the satisfaction of psychological needs. As per this definition, adolescents necessitate effort, power, energy, and motivation to address their psychological needs. Similarly, heightened motivation is anticipated when an adolescent’s actions yield positive outcomes, indicative of their needs being satisfied (Chen and Hypnar, 2015). Notably, in countries like China, where the one-child policy is implemented, adolescents reportedly face unsatisfied psychological needs due to limited opportunities for interaction with siblings and peers during leisure time (Qiang-hui, 2011). Given the positive correlation between intrinsic motivation and competence (Kalajas-Tilga et al., 2020), it is expected that these adolescents will exhibit diminished motivation levels.

On the other hand, the challenges faced by adolescents in meeting their psychological needs may stem from inadequate support for the adolescent’s autonomy by their parents. This form of struggle is expected to diminish self-control, leading to a depletion of motivational strength (Baumeister et al., 2007; Hagger et al., 2010). Considering that, according to Self-Determination Theory (SDT), intrinsic motivation stands out as the most autonomous form of motivation (Ryan and Deci, 2000), this scenario is poised to curtail intrinsic motivation by impeding self-regulation. When a person cannot or does not exert control over themselves, automatic adherence to habitual behaviors is anticipated (Tiffany, 1990).

In the absence of an adolescent’s self-control, it can be argued that they will exhibit normal, typical, or automatic behaviors rather than the actions that motivate and fulfill them, consequently leading to a decrease in intrinsic motivation and an increase in amotivation. It could be argued that in the absence of self-control in an adolescent, they may exhibit normal, typical, or automatic behaviors instead of engaging in actions that motivate and satisfy them, thereby affecting their level of intrinsic motivation and amotivation. Highlighting the mediating role of psychological need satisfaction and intrinsic motivation in the relationship between perceived parental autonomy support and behavior performance, the research conducted by Kalajas-Tilga et al. (2020) reveals the indirect connections between these concepts. In conclusion, the impact of parental autonomy support on intrinsic motivation is indirectly shaped by its influence on psychological need satisfaction and self-control. Halfmann and Rieger (2019) found that attitudes not supportive of autonomy, such as social pressure, negatively affect autonomy and competence. This finding supports the mentioned perspective. In that study, social pressure was specifically noted to lead to self-control failures, associated with a decrease in competence. Similarly, Tangney et al. (2004) associated insecure attachment with lower self-control. The research findings indicating a positive relationship between self-control and motivation align with these study results (Muraven and Slessareva, 2003). In conclusion, it can be stated that unsupportive, controlling, or punitive parenting attitudes in daily life fail to satisfy adolescents’ psychological needs and weaken their self-control. Consequently, this diminishes intrinsic motivation and increases the level of amotivation.

## Conclusion

When examining the key findings of the study, crucial results emerge for intervention programs aimed at increasing adolescents’ motivation and providing parental autonomy support. Especially, the finding that the emphasized psychological needs and self-control in SDT have an indirect impact on the relationship between parental autonomy-supportive attitudes and motivation adds new insight to the ongoing discussions in the literature regarding variables with an indirect effect on motivation. While the literature on motivation predominantly focuses on physical activity performance (Wilson et al., 2004; Vierling et al., 2007; Zhang et al., 2011), this study directs attention to the factors of parental autonomy support, satisfaction of psychological needs, and the concept of self-control that influence motivation.

Numerous studies suggest that adolescents particularly desire support for their feelings of autonomy, such as status or respect, from their parents, and that when this support is provided, their learning, i.e., academic motivation, is strongly influenced (Crone and Dahl, 2012; Blakemore and Mills, 2014; Telzer, 2016). Specifically, considering that academic motivation levels of 7th-grade students are significantly higher than those of 9th-grade students, and motivation of 9th-grade students is higher than that of 11th-grade students (Yeung and McInerney, 2005), the significance of factors such as parental autonomy support influencing motivation during adolescence becomes more apparent. The gradual decline in motivation during adolescence underscores the need to investigate factors influencing this change. Future research can support studies specifically focused on changes in academic motivation during adolescence and the factors influencing this change.

This research demonstrates that adolescents perceiving autonomy support from their parents can carry out their behaviors more autonomously, feel more competent in executing these behaviors, and perceive themselves as more successful in building relationships with others. This finding highlights the importance of considering the role of parents in interventions for adolescents facing difficulties due to the failure to satisfy their psychological needs. Subsequent studies can contribute to this perspective by testing the relationship between these two variables. Moreover, the finding of the association between psychological needs and self-control in this research contributes to programs or research aiming to enhance adolescents’ self-control skills. Especially, interventions addressing psychological needs are likely to be more effective in improving self-control skills.

In particular, the revelation in this article that self-control is associated with types of motivation will contribute to research on complaints associated with demotivation (e.g., depression, anxiety, stress). This perspective aligns with the fundamental views of SDT. According to SDT, control sources perceived from parents are internalized over time. As individuals internalize control sources, they experience more autonomy in their own worlds (Ryan and Deci, 2000). Individuals who experience autonomy in their own worlds are expected to achieve high levels of motivation and success by regulating themselves and controlling their behaviors (Vierling et al., 2007; Zhang et al., 2011; Wang, 2017).

The findings of this study may provide insights for parents, educators, administrators, and policymakers. These individuals can enhance adolescents’ autonomy, relatedness, and competence by increasing their opportunities for choice, developing relationships with peers and family, setting expectations proportional to their performance, and increasing their competence by providing positive and informative feedback. Meeting psychological needs enhances adolescents’ self-control skills, not only increasing motivation levels at school or within the family but also improving their well-being levels (Niemiec and Ryan, 2009; Chang et al., 2017). Acknowledging this situation, individuals effective in adolescents’ lives are considered to contribute to their healthy development. Unsupportive, overly controlling, or punitive relationships in daily life can weaken individuals’ self-control, leading to decreased motivation levels and an increase in problematic behaviors (Halfmann and Rieger, 2019).

Lastly, highlighting the indirect impact of parental autonomy-supportive attitudes, psychological needs, and self-control on motivation, this view broadens the understanding of what influences motivation beyond direct factors like physical activity performance. This expanded understanding allows researchers to develop more comprehensive models of motivation. The recognition of parents’ autonomy-supportive attitudes as a factor influencing motivation underlines the importance of familial and social environments in shaping individual motivation. This insight may inform interventions aimed at fostering a supportive environment and improving parenting strategies that enhance motivation offered by psychologists. Understanding that psychological needs and self-control mediate the relationship between parental autonomy support and motivation provides researchers with specific variables to target in interventions. By focusing on enhancing psychological needs satisfaction and self-control, mental health professionals can potentially amplify the positive effects of autonomy-supportive parenting on motivation. This perspective can integrate insights from Self-Determination Theory (SDT) and other related theories into the study of motivation. By synthesizing various theoretical perspectives, researchers can develop a more nuanced understanding of motivational processes, facilitating interdisciplinary collaboration and theory building. The findings from this research are expected to contribute to practical applications in fields such as education, sport psychology, and clinical psychology. Educators, coaches, and therapists can use this knowledge to design interventions that promote autonomy support, psychological well-being and self-control, and thus increase motivation and overall well-being in their field.

### Limitation and future implications

This study has some limitations. The study solely collected quantitative data from the study group, and these data were not gathered at different time points. The cross-sectional design used in this study prevents making causal inferences. Future studies could test reverse causality with a longitudinal design. Moreover, manipulating the sequence of variables might augment comprehension of the interrelations among these factors. Another limitation could be the reliance on participants’ subjective assessments. While subjective judgments are customary in appraising personal thoughts and emotional experiences, the integration of additional sources could enhance data reliability. Subsequent investigations might integrate opinions from teachers, parents, or peers to furnish supplementary insights and bolster data reliability, addressing the potential influence of social desirability bias on adolescent responses. In addition, variables such as gender and grade level were not analyzed in this study. It is recommended to include such variables in future studies.

One more limitation arises from the exclusive focus on individuals from a comparable culture and developmental stage. Future studies might strive to diversify data collection by incorporating both qualitative and quantitative measures at different junctures, encompassing individuals from diverse cultural backgrounds and developmental stages to scrutinize potential variable effects. Despite these limitations, the study boasts notable strengths. Notably, it contributes to unraveling the intricate connections between adolescents’ autonomy support, psychological need satisfaction, self-control, and various motivational types. Such insights not only enrich our comprehension of the normative processes during the tumultuous adolescent developmental phase but also offer valuable inputs for devising and implementing psychoeducational interventions pertaining to these variables.

## Data availability statement

The original contributions presented in the study are included in the article/supplementary material, further inquiries can be directed to the corresponding author.

## Ethics statement

The studies involving humans were approved by Erzincan Binali Yıldırım University scientific research and publication ethics committee https://etikkurul.ebyu.edu.tr/insan-arastirmalari-etik-kurulu/egitim-bilimleri/basvuru/. The studies were conducted in accordance with the local legislation and institutional requirements. Written informed consent for participation in this study was provided by the participants' legal guardians/next of kin.

## Author contributions

OÇ: Writing – review & editing.
